# Sub-Lethal Toxicity of Bifenthrin and Acetamiprid Through Dietary Trophic Route: Effects on the Foraging Activity, Social Interactions, and Longevity of *Apis mellifera* L.

**DOI:** 10.3390/insects17020141

**Published:** 2026-01-26

**Authors:** Muhammad Usman Yousuf, Muhammad Anjum Aqueel, Shams Ul Islam, Sohail Akhtar, Mirza Naveed Shahzad, Rohma Amal, Muhammad Saqib, Aiman Hina, Nyasha J. Kavhiza, Mishal Subhan

**Affiliations:** 1 Department of Entomology, Faculty of Agriculture and Environment, The Islamia University of Bahawalpur, Bahawalpur 63100, Pakistan; 2 Division of Biology, Silwood Park Campus, Imperial College London, Ascot SL5 7PY, UK; 3 Honeybee Lab, Department of Biology, University of Oxford, Oxford OX1 3EL, UK; 4 Department of Statistics, University of Gujrat, Gujrat 50700, Pakistan; 5 Department of Agronomy, Faculty of Agriculture and Environment, The Islamia University of Bahawalpur, Bahawalpur 63100, Pakistan; 6 Ministry of Agriculture (MOA) National Centre for Soybean Improvement, State Key Laboratory for Crop Genetics and Germplasm Enhancement, Nanjing Agricultural University, Nanjing 210095, China; aimanhina@yahoo.com; 7 Department of Environmental Management, Institute of Environmental Engineering, RUDN University, 6 Miklukho-Maklaya St., 117198 Moscow, Russia; 8 Department of Microbiology and Molecular Genetics, The Women University Multan, Multan 66000, Pakistan

**Keywords:** *Apis mellifera* L., trophic exposure, sub-lethal insecticide, bifenthrin, acetamiprid, foraging behavior

## Abstract

*Apis mellifera* L. is an important pollinator of both wild and domesticated crop plants, thus greatly contributing to plant biodiversity and commercial agriculture. However, in field conditions, honey bees remain exposed to different pesticides which ultimately affect colony health parameters and their associated ecological services. In the current study, the individual toxicities of sub-lethal doses of two distinct insecticides (bifenthrin and acetamiprid) belonging to different groups (pyrethroid and neonicotinoid) were assessed against the foraging activity, social interactions, and longevity of *A. mellifera*. The bees were exposed to individual doses of both insecticides via the dietary trophic route through contaminated pollen and nectar under natural field conditions. Sunflower crop (Hysun-33) was sown at nine different sites with an isolation distance of 3 km, and was treated with different doses (1/2, 1/4, 1/10, and 1/20 of the recommended field doses) of both insecticides. However, the untreated control crop plots were not subjected to any chemical treatments (bee colonies received no insecticide, and served as the baseline for making comparison). Twenty-seven bee colonies were introduced in these sites after seven days of treatment applications. Significant differences were observed in the foraging activity of *A. mellifera* (including bees going out from the hive, returning foragers, and those carrying pollens). The fecundity, adult longevity, and social behaviors like trophallaxis and antennation were significantly lower in bees exposed to higher individual insecticidal concentrations. However, the hatching duration, larval duration, and pupal duration were not affected by the tested insecticidal treatments. Overall, these findings demonstrate that the dietary trophic exposure of sub-lethal doses of insecticides compromised colony activities, which is indeed a matter of concern regarding the existing pesticide application methods in different agro-ecosystems. Such impacts may ultimately impair the survival of colonies, particularly when bees remain exposed to these chemicals over an extended period of time. Therefore, future studies must consider the pesticide application techniques and their application timing to mitigate the direct and indirect negative impacts of pesticides on pollinators.

## Simple Summary

Honey bees (*Apis mellifera* L.) play a vital role in the pollination of different crop plants but remain exposed to lethal and sub-lethal doses of pesticides which are used in different farming systems. This study investigated how the indirect individual dietary trophic exposure of two commonly used insecticides, bifenthrin and acetamiprid, belonging to two different groups (pyrethroid and neonicotinoid), affect bee colonies. Sunflower fields (Hysun-33) were subjected to different sub-lethal doses of these insecticides. After seven days of individual insecticidal treatments, twenty-seven bee colonies were introduced at nine different sites in these fields. The isolation distance between each site was not less than 3 km. The colonies introduced in the insecticide-free plots were considered as untreated control (T0). The results revealed that the bee colonies exposed to higher individual doses of insecticides exhibited less foraging activity, weaker social behaviors such as food sharing and communication, and shorter lifespans compared to the untreated control colonies. However, the growth durations of different immature stages of bees (egg, larva, and pupa) were not affected by the individual exposure of these insecticides. These findings suggest that even small amounts of pesticides taken through contaminated food can disturb the normal behavior and health of honey bee colonies. The results highlighted the need to use pesticides more precisely, especially by adjusting their timing and application methods, to protect pollinators that serve as an essential component of crop production and ecological sustainability.

## 1. Introduction

*Apis mellifera* L. contributes significantly in the pollination of over 70% of the crop species worldwide [1,2]. Their pollination services not only support plant biodiversity but also improve crop growth and yield. Bees are an important contributor of small- and large-scale commercial farming systems, adding billions of dollars annually to global agricultural economies [1,3]. The market value of a vast number of high-valued crops depends on their pollination services, which makes bees an essential component of different food production systems and rural livelihoods [4,5]. However, for the last few years, bees are experiencing severe population declines, which not only compromises their ecosystem services but also poses serious implications for food and nutritional security across the globe [6,7].

*Apis mellifera* is an important pollinator, which spends most of its time in vegetation to find different food resources for efficient brood development [8]. The health and development of a bee colony is maintained through trophallaxis, antennation, and division of labor, which not only regulate the flow of resources and information, but also make colonies more resilient against different environmental changes [9]. Different biotic and abiotic stressors such as habitat destruction, pathogen invasion, and pesticidal exposure pose a serious threat for bee colonies and their associated pollination services [10,11]. Although the causes of honey bee decline may be complex and interrelated, the widespread use of different pesticides in agro-ecosystems has been considered a potential contributing factor [12,13].

Insecticides, fungicides, and herbicides are mostly used as pesticides to safeguard crops from different biotic stressors. However, their widespread use has also contaminated the agricultural landscapes, which poses serious threats for pollinators and other non-target organisms [14,15]. Synthetic insecticides such as bifenthrin, belonging to the group of pyrethroids, are preferred in different agro-ecosystems due to their quick mode of action against different insect pests [16]. Similarly, acetamiprid, a neonicotinoid, binds with nicotinic acetylcholine receptors of the insect nervous system, leads to overstimulation of the nervous system, and compromises insect vital body functions, leading to the death of the organism [17,18]. However, despite being regarded as less toxic in comparison to other neonicotinoids, sub-lethal exposure of acetamiprid is also capable of disrupting learning, olfactory memory, and social recognition in honey bees [19]. It has been reported that the systemic properties of most of the modern-day insecticides facilitate their translocation to nectar and pollen, which might later contaminate honey bees, mainly through feeding [20].

Honey bees, mainly foragers, are more prone to infield pesticidal exposure, due to their foraging flights, compared to in-hive nurse bees [21]. The bees endure pesticides in various ways, mainly through contaminated nectar, pollen, water, and other hive material [22]. Moreover, trophic transfer via diet occurs when the accumulated pesticide residues in food sources impact the entire colony [23]. Pesticide residues passed through trophallaxis and jelly induce sub-lethal effects on the health and reproduction of bees [24,25]. The exposure to sub-lethal doses of pesticides usually does not lead to immediate death, but affects certain physiological and behavioral aspects of bees, which results in decreased foraging efficiency, navigation, social homeostasis, and immune competence, thus weakening the survivability of a colony [26,27]. Moreover, low or sub-lethal concentrations of bifenthrin and other insecticides interfere with learning, memory, and social behaviors like trophallaxis and grooming of honey bees. These disruptions influence the foraging patterns and intra-colony communication that are vital for the homeostasis of the colony [28,29]. Antennal contact, trophallaxis, and grooming all play important roles in communication and exchange of information, food and pheromones. These behaviors guarantee integration in the colony and adaptive responses against stressors [30]. Sub-lethal exposure to pesticides also impairs queen physiology, decreases its fecundity, and changes pheromone synthesis, which has an adverse impact on the reproduction of the colony and behavior of the worker bees [31,32,33].

Pesticidal exposure also reduces brood viability by compromising the quality of the brood feed, which results in production of a smaller and less viable worker population [24,34]. Decreased fecundity and brood survival of queens not only reduce the growth of colonies, the size of workforce, and the output of honey, but also endangers the sustainability of colonies and pollination services [35,36,37]. Reduced reproduction and colony strength limits pollination services, underscoring the need to assess the sub-lethal effects of pesticides on honey bees [11]. Field-based experiments that incorporate environmental factors and realistic exposure pathways provide more precise estimates of how pesticides influence colony performance and social behavior of honey bees [38].

Most of the previous literature largely remained focused on exploring the impacts of direct contact or acute toxicity of pesticides on honey bees. However, the trophic exposure route of pesticides (via contaminated nectar or pollen, or through their food) remains largely underexplored, particularly concerning its effects on foraging activity, social behavior, and bee longevity [24]. This study introduces a novel perspective in honey bee toxicology by examining the sub-lethal trophic (dietary) pathway of insecticide exposure instead of conventional toxicity routes (direct or surface residual). It investigated the trophic effects of sub-lethal concentrations of two popular insecticides, i.e., bifenthrin (a pyrethroid) and acetamiprid (a neonicotinoid) on *A. mellifera* in field-relevant conditions. The work is distinguished from its counterparts by its field-scale experimental design, integration of multiple behavioral and social endpoints (foraging activity, trophallaxis, antennation, and longevity), and the comparative assessment of two insecticide classes. These findings enhance knowledge of how long-term, low-level dietary exposure of insecticides can influence colony dynamics, individual bee behavior, and ultimately pollination performance and colony sustainability.

## 2. Materials and Methods

### 2.1. Experimental Design and Colony Setup

The current experiment required meticulous observations and therefore involved an independent designated field area which can be controlled over certain variables as per study requirements. The selected fields fulfilling the above requirements belong to the Apiculture Research Field, Department of Entomology, The Islamia University of Bahawalpur (Punjab, Pakistan). The study also involved twenty-seven homogenized, healthy, proportionately similar, and disease- and pest-free bee colonies (*A. mellifera ligustica*) with fertile queen bees. The bee colonies were kept in hives and equally aligned regarding adult bee population, colony strength, and brood area. All the queens, obtained from a single source colony, were fertile and of similar age. This standardization minimized the chances of variability among colonies and ensured that the observed effects were primarily related to insecticide exposure rather than initial colony differences [39]. Initially, the bee colonies were maintained at the Apiculture Research Area, The Islamia University of Bahawalpur. During this colony maintenance period, the foraging sources within a 3 km vicinity of the Apiculture Research Area were not subjected to any chemical treatments. The hive chambers were checked regularly to ensure that bees were performing in the best possible way as a healthy colony.

**Figure 1:**
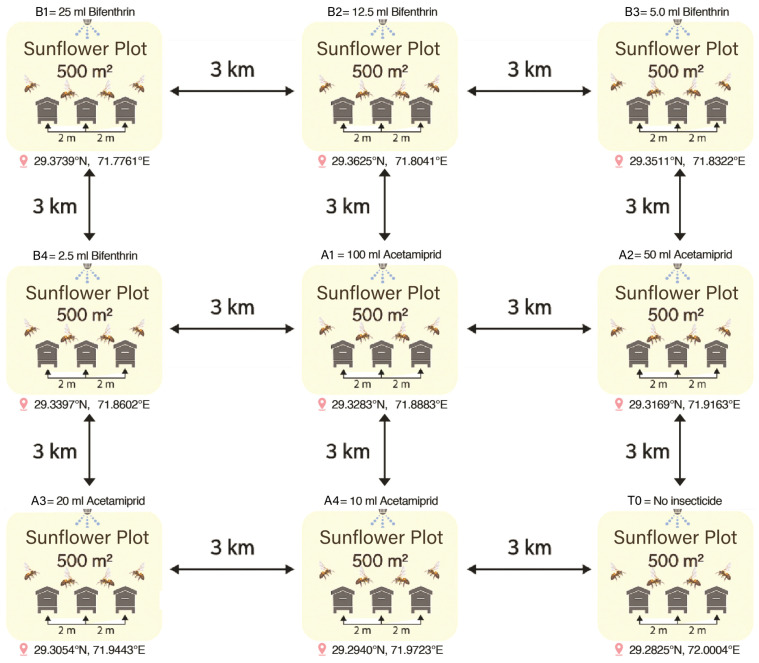
Systematic diagram showing the placement of 27 bee colonies and sunflower crop fields. Each plot (500 m^2^) was separated from the others by a minimum distance of 3 km, while three bee colonies were placed within each sunflower field at 2 m spacing from each other.

Sunflower (Hysun-33) was planted in the last week of January 2023 at nine different locations (plots) (29.3739° N, 71.7761° E; 29.3625° N, 71.8041° E; 29.3511° N, 71.8322° E; 29.3397° N, 71.8602° E; 29.3283° N, 71.8883° E; 29.3169° N, 71.9163° E; 29.3054° N, 71.9443° E; 29.2940° N, 71.9723° E; 29.2825° N, 72.0004° E) of the Apiculture Research Field, each with an area of 500 m^2^. The crop was subjected to all required agronomic practices to maintain a healthy stand. The area (500 m^2^) was deemed enough to rear three honey bee colonies [40]. The foraging range of honey bees is reported to be 1.5–2 km [41,42]; therefore, each crop plot was kept 3 km apart to prevent foraging overlap among bee colonies (Figure 1). Sunflower flowering commenced in the second week of March 2023, marking the initiation of the experimental phase. The average temperature of the experimental site ranged between 28 and 34 °C, with relative humidity around 45–55%. No significant rainfall was recorded during the experimental period, providing favorable conditions for foraging activity. Moreover, before the execution of the experiment, meetings were organized with farmers to ensure that no insecticides were applied on plots near the experimental vicinity. Spraying of both insecticides was conducted according to a pre-planned schedule on the 3rd, 4th, and 5th of April 2023, with three plots treated each day. The data from each plot was collected after similar time intervals of insecticidal applications to avoid any experimental error.

### 2.2. Preparation of Sub-Lethal Doses of Tested Insecticides

The commercial formulations of Bifenthrin 10 EC (Talstar^®^ 10 EC, FMC Corporation, Philadelphia, PA, USA) and Acetamiprid 20 SP (Acelan^®^ 20 SP, FMC Corporation, Philadelphia, PA, USA) were obtained from a local pesticide market. To simulate oral trophic exposure via contaminated nectar and pollen, bee colonies were introduced in the insecticide-treated sunflower plots after one week of spraying to avoid any direct contact with insecticide treated plant surfaces. In this case, bees could only be exposed to insecticidal treatments via contaminated pollen or nectar through the dietary pathway [43,44]. The insecticides were applied on the sunflower crop at the flowering stage using a knapsack sprayer, following standard field application protocols [45]. Sub-lethal doses of insecticides were prepared by diluting the recommended field rates of Bifenthrin (50 mL/500 m^2^) and Acetamiprid (200 mL/500 m^2^) in water. Four treatments for each insecticide were prepared: one-half (B1 and A1), one-quarter (B2 and A2), one-tenth (B3 and A3), and one-twentieth (B4 and A4) of the recommended doses. A control treatment (T0) with no insecticide was also included. The specific amounts of insecticides applied per 500 m^2^ plot are provided in Table 1.

### 2.3. Foraging Activity

The foraging activity of the bees was determined by visual observation, i.e., by counting the bees present in the hive following the method adopted by Islam et al. [39]. The numbers of bees going out of the hive for foraging [46], returning back to their hives [47], and those carrying pollen on their legs, were recorded for 5 min [48] between 8:00 to 10:00 a.m. The mentioned time period corresponds to peak foraging activity of *A. mellifera*, as documented in previous studies [49,50].

### 2.4. Social Interaction

In the social behavior experiments, two forms of bee behaviors, i.e., antennation and trophallaxis, were assessed. The hives were regularly monitored with high-pixel cameras, Go-Pro Hero (CHDRB-101-CN), GoPro, Inc., San Mateo, CA, USA. The cameras were installed at a fixed and identical position facing the central brood frame of all colonies, where social interactions are most frequent. The camera setup was standardized across all beehives and remained unchanged throughout the observation period to ensure minimal disturbance among colonies. A non-intrusive low-intensity light was used to light up the hive so that the camera could get high-quality footage without disturbing the bees. The source of light was also placed strategically to avoid any direct contact with the bees and so as to reduce any kind of stress or behavioral alterations [51]. Videos were recorded for a total of 30 min, divided into three 10 min sessions. Antennation and trophallaxis are rapid and frequent behaviors, and therefore can be reliably quantified within such short observation windows [39,52,53,54]. The recorded footage was subsequently analyzed for obtaining precise results.

### 2.5. Longevity

To measure dietary trophic effects in all the experimental colonies, empty frames containing wax only were placed in the center of the hive, in which the number of eggs laid by the queen bee were counted [55]. The data regarding egg counts was taken for 5 days. After that, the counted number of eggs was labeled to observe their successful transformation into the pupae and locate broods in the hives. The eggs laid by the queen were tagged and observed for egg hatching period, larval period, and pupal period. Once the adult workers emerged, they were marked using POSCA color marker (non-toxic marker) (POSCA PC-5M, Mitsubishi Pencil Co., Ltd., Boulogne-Billancourt Cedex, Boulogne-Billancourt, France) on their thorax [49,56], and monitored daily in the morning at 10 am, until they died or were found missing. The identified bees were counted and the adult longevity data for all tested treatments were recorded [57].

### 2.6. Statistical Analysis

Statistical analyses were performed using R software (version 4.3.0). The data regarding experimental parameters, such as foraging activity (bees left, returning bees, pollen collection), and longevity indicators (fecundity, hatching period, larval period, pupal period, and adult lifespan were analyzed under Randomized Complete Block Design (RCBD), while one-way analysis of variance (ANOVA) under Complete Randomized Design (CRD) was applied for data regarding social interactions (trophallaxis frequency and antennation frequency). The significance of differences between treatment means was established through the Least Significant Difference (LSD) test at 5% significance level. Data was pre-tested to ensure that the data were normally distributed (Shapiro–Wilk test) and had equal variances (Levene’s test). There was no need for data transformation, as the assumptions were met. Pearson correlation analysis between trophallaxis and antennation frequencies was performed in R to assess the strength and direction of their linear relationship. To visualize and compare the treatment effects, all graphs were generated using the ggplot2 package (version 3.5.1) and dplyr package (version 1.1.4), while for correlation analysis, the corrplot package (version 0.95) was used in the same software.

## 3. Results

### 3.1. Foraging Activities

#### 3.1.1. Bees Leaving Hive

**Figure 2:**
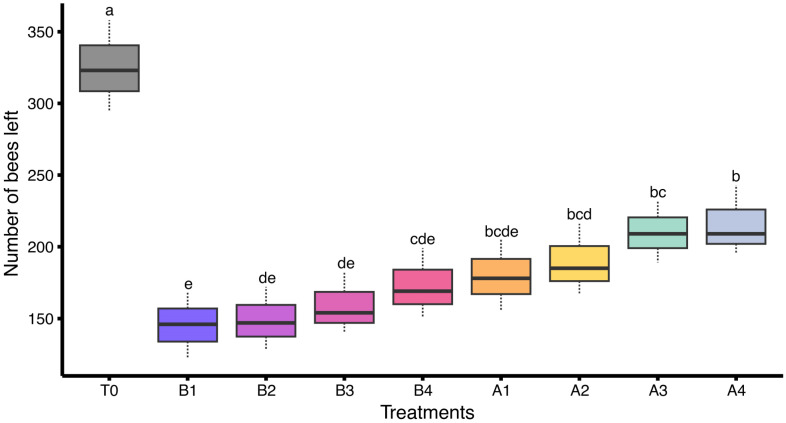
Number of bees leaving the hive in colonies subjected to different insecticides under field conditions (*n* = 3 colonies per treatment). Different letters indicate statistically significant differences among treatments based on LSD test at α = 0.05. The dashed lines (whiskers) above and below of each box represent data variability.

A statistically significant difference was observed in the number of bees leaving the hive among colonies exposed to different insecticide treatments under field conditions (F_(8,18)_ = 15.1, *p* < 0.05). The highest bee population leaving the hive was recorded in the control group (T0: 325.00 ± 18.50 bees). Colonies treated with A4 (215.67 ± 14.25 bees) and A3 (210.00 ± 12.42 bees) showed moderate numbers of bees leaving the hive, whereas lower populations were observed in A2 (189.33 ± 14.31 bees), A1 (179.67 ± 14.17 bees), and B4 (173.00 ± 14.00 bees). The lowest bee populations leaving the hive were recorded in colonies subjected to B3 (159.00 ± 12.66 bees), B2 (149.00 ± 12.74 bees), and B1 (145.33 ± 13.28 bees). These results indicate a clear dose-dependent reduction in foraging activity with increasing insecticide exposure (Figure 2).

#### 3.1.2. Bees Returning to the Hive

**Figure 3:**
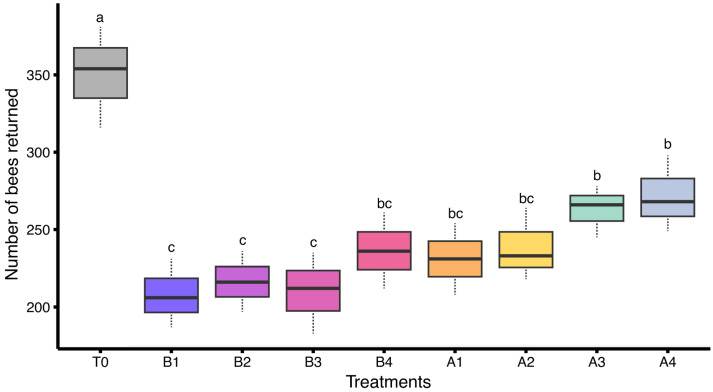
Number of bees returning to the hive in colonies treated with various insecticides under field conditions (*n* = 3 colonies per treatment). Different letters indicate statistically significant differences among treatments based on LSD test at α = 0.05. The dashed lines (whiskers) above and below of each box represent data variability.

A statistically significant difference was observed in the number of bees returning to the hive among colonies exposed to different insecticide treatments under field conditions (F_(8,18)_ = 10.3, *p* < 0.05). The highest number of returning bees was recorded in the control group (T0), with a mean of 350.33 ± 18.85 bees, reflecting the absence of chemical stress. Colonies treated with A4 (271.67 ± 14.26) and A3 (263.00 ± 9.64) showed moderate return numbers, while slightly lower numbers were observed in A2 (238.33 ± 13.54), B4 (236.33 ± 14.15), and A1 (231.00 ± 13.28). The lowest returning bee populations were recorded in colonies exposed to B2 (216.33 ± 11.26 bees), B3 (210.00 ± 15.04 bees), and B1 (208.00 ± 12.74 bees). This trend reflects reduced foraging efficiency and potential navigational impairment caused by insecticidal stress (Figure 3).

#### 3.1.3. Bees Containing Pollens

**Figure 4:**
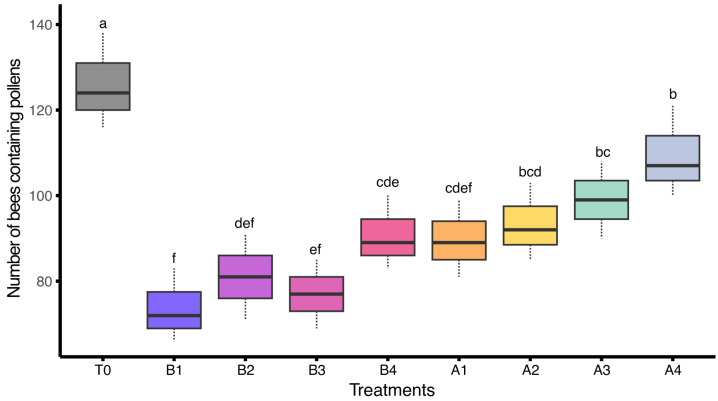
Number of bees carrying pollens, treated with various insecticides under field conditions (*n* = 3 colonies per treatment). Different letters indicate statistically significant differences among treatments based on LSD test at α = 0.05. The dashed lines (whiskers) above and below of each box represent data variability.

Similarly, the number of bees exposed to different insecticidal treatments under field conditions showed significant differences regarding bees that carried pollens in their legs (F_(8,18)_ = 9.25, *p* < 0.05). The highest number of bees carrying pollens was recorded in the control group (T0) with a mean of 126.00 ± 6.43, indicating optimal foraging performance in the absence of chemical exposure. Colonies treated with A4 (109.33 ± 6.17) and A3 (99.00 ± 5.20) showed moderate pollen collection, while slightly lower values were recorded in A2 (93.33 ± 5.24), B4 (90.67 ± 4.98), and A1 (89.67 ± 5.21). The lowest number of bees carrying pollens was recorded in B2 (81.00 ± 5.77), B3 (77.00 ± 4.62), and B1 (73.67 ± 4.98). This pattern suggests that sub-lethal insecticide exposure negatively affects resource collection and foraging motivation (Figure 4).

### 3.2. Social Interactions

#### 3.2.1. Trophallaxis

**Figure 5:**
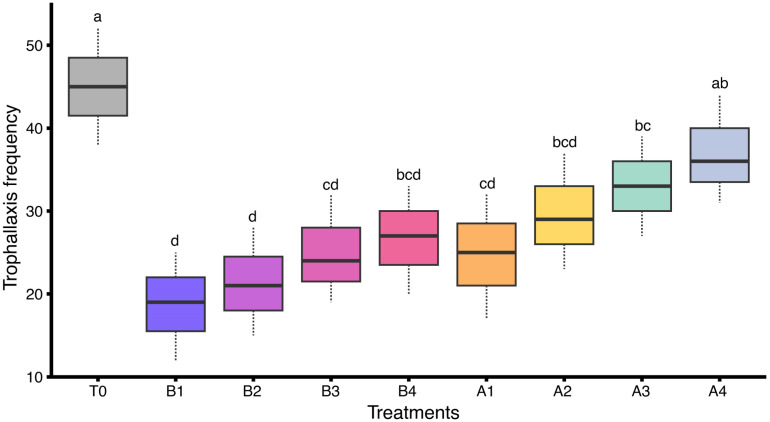
Trophallaxis frequency in honey bee colonies treated with various insecticides under field conditions (*n* = 3 colonies per treatment). Different letters indicate statistically significant differences among treatments based on LSD test at α = 0.05. The dashed lines (whiskers) above and below of each box represent data variability.

A statistically significant difference was observed in trophallaxis behavior of honey bee colonies subjected to different insecticide treatments under field conditions (F_(8,18)_ = 4.53, *p* < 0.05). The control group (T0) had the highest mean frequency (45.00 ± 4.04), indicating active social food-sharing in the absence of chemical exposure. Moderate activity was observed in A4 (37.00 ± 3.79) and A3 (33.00 ± 3.46), while lower values were recorded in A2 (29.67 ± 4.06), B4 (26.67 ± 3.76), B3 (25.00 ± 3.79), and A1 (24.67 ± 4.33). The lowest trophallaxis frequencies were found in B2 (21.33 ± 3.76) and B1 (18.67 ± 3.76). This indicates that insecticide stress may impair trophallaxis behavior and intra-colony communication (Figure 5).

#### 3.2.2. Antennation

**Figure 6:**
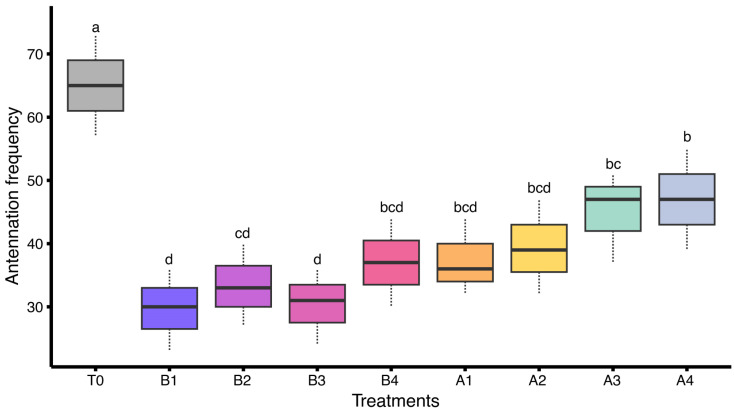
Antennation frequency in honey bee colonies treated with various insecticides under field conditions (*n* = 3 colonies per treatment). Different letters indicate statistically significant differences among treatments based on LSD test at α = 0.05. The dashed lines (whiskers) above and below of each box represent data variability.

Similarly, a statistically significant difference was observed in antennation behavior of honey bee colonies subjected to various insecticide treatments under field conditions (F_(8,18)_ = 7.29, *p* < 0.05). The highest frequency was recorded in the control group (T0: 65.00 ± 4.62), reflecting strong social interaction. Moderate levels were observed in A4 (47.00 ± 4.62) and A3 (45.00 ± 4.16), followed by lower activity in A2 (39.33 ± 4.33), A1 (37.33 ± 3.53), and T4 (37.00 ± 4.04). Reduced antennation was seen in B2 (33.33 ± 3.76), B3 (30.33 ± 3.48), and the lowest was observed in B1 (29.67 ± 3.76). These results support the conclusion that sub-lethal insecticide exposure reduces social responsiveness within the colony (Figure 6).

### 3.3. Pearson’s Correlation

The Pearson correlation between two critical social behaviors of *A. mellifera*—trophallaxis (food-sharing behavior) and antennation (antenna-based social contact)—was observed. The Pearson correlation coefficient (r = 0.94) indicated a very strong positive relationship, meaning that as trophallaxis increased, antennation also tended to increase, and vice versa. The statistical significance of this correlation was confirmed by the *p*-value < 0.001, indicating a highly significant association. These findings suggested that both behaviors were closely interlinked and potentially influenced by shared stressors, such as sub-lethal insecticidal exposure. Therefore, disruptions in either of these behaviors could serve as early indicators of compromised colony social dynamics under indirect insecticide stress. This highlights that both social behaviors are closely interlinked and can serve as sensitive indicators of colony stress under sub-lethal insecticidal exposure.

### 3.4. Longevity and Reproduction

A statistically significant difference was observed in fecundity and adult bee lifespan of honey bees belonging to colonies which were subjected to different insecticide treatments under field conditions. Fecundity varied significantly across treatments (F_(8,18)_ = 5.6, *p* < 0.01), with the highest egg-laying recorded in the control group (T0: 250.00 ± 12.10), followed by A4 (214.00 ± 11.24) and A3 (203.67 ± 11.78). The lowest fecundity was observed in B1 (169.33 ± 8.37), indicating reduced reproductive output under that treatment. Similarly, adult bee lifespan differed significantly (F_(8,18)_ = 11.0, *p* < 0.01), with the longest lifespan observed in T0 (41.67 ± 0.33 days), followed closely by B4 (41.33 ± 0.67) and A4 (40.67 ± 0.67). The shortest lifespans were observed in B1 and B2 (36.33 ± 0.88 and 36.33 ± 0.33 days, respectively), reflecting the weaker support provided by these treatments for bee longevity. The results further showed slight but statistically non-significant variations regarding the hatching period (F = 1.25, *p* = 0.3276), larval period (F = 1.29, *p* = 0.308), or pupal period (F = 0.85, *p* = 0.5705), suggesting relatively uniform developmental durations across all tested treatments during these stages. However, numerical differences were noted: A2 had the shortest larval period (5.00 ± 0.00 days), while T0 and A4 had the longest (6.00 ± 0.00 days). Similarly, the pupal period remained at 12.00 ± 0.00 days in T0 and A4, whereas slightly shorter durations were recorded in other treatments (Table 2).

## 4. Discussion

The current study introduces a novel perspective in honey bee toxicology by examining the sub-lethal trophic (dietary) pathway of insecticidal exposure instead of conventional toxicity routes (direct and surface residual). The current research assessed the individual dietary trophic effect of bifenthrin and acetamiprid on some colony parameters such as foraging behavior, social interaction, and longevity of *A. mellifera* under field conditions.

The foraging activities of bees showed a consistent decline when colonies were exposed to a higher concentration of insecticides, especially bifenthrin at 1/2 (B1) and 1/4 (B2). The control group (T0) showed the best foraging activities, with the maximum number of bees returning to hives, suggesting optimal colony performance in the absence of chemical stress. The current results were supported by the findings of Henry et al. [35], who proved that application of thiamethoxam decreased the capability of the foragers to come back to the hive, which they explained might be due to the loss of navigation as a results of neurotoxic effect of applied chemical. Similarly, Decourtye et al. [58] reported that application of sub-lethal concentrations of deltamethrin and imidacloprid influenced the learning and orientation of bees, resulting in reduced foraging success. Moreover, a moderate decline in foraging activity was also observed at lower insecticide doses (B3–A4), indicating that chronic trophic exposure, even at reduced concentrations, may influence foraging efficiency over time rather than causing immediate disruption. This aligns with the work of Tosi and Nieh [59], who pointed out that flupyradifurone, another systemic insecticide, caused long-term behavioral interference in bees even at low doses. Similarly, Mustard et al. [60] reported that honey bees exposed to different pesticide-treated nectars and pollens remain confused in perceiving floral odors, which significantly compromises (up to 40%) their foraging activities. This might be attributed to the fact that pesticidal exposure alters the neural pathways linked with associative learning, making it difficult for bees to find food resources and locate earlier valuable floral sources [61]. The current results were further supported by Piiroinen and Goulson [62], who reported that chronic exposure to new-chemistry insecticides delays learning speed and cognitive processing in bumblebees and honey bees. These learning delays are mostly associated with weaker colony stability and reduced foraging activities. Moreover, the time span for recording these observations varied in different studies. but a two-hour window in the morning, as mentioned in current study, usually represents the peak foraging time interval of honey bees in different ecosystems [41,47,50,63]. Moreover, foragers possess the ability to remember the specific time of the day during which they found abundant food resources and later prefer that time to initiate their foraging activities [64]. However, the usual foraging interval at a specific feeding site is no more than five minutes [65].

The collection of pollens is a direct indicator of the performance of foraging and nutritional conditions of colonies. Bees exposed to higher concentrations of bifenthrin (B1, B2) and acetamiprid (A1) collected less pollen than the untreated control. Such declines have also been reported by Singla et al. [66], who noted that the neonicotinoids decreased the quantity of pollen on returning foragers because of motor deficiencies and reduced floral attraction. In addition, social behaviors such as trophallaxis and antennation were reduced in pesticide-treated colonies, suggesting potential disruption of intra-colony communication rather than complete behavioral suppression. Trophallaxis, essential for food sharing and pheromonal communication, was highest in T0 but decreased in proportion against the rising level of insecticide exposure, especially in B1 and B2. These findings were consistent with Murphree [67], who reported that neonicotinoids interfered with the recognition of colony mates and reduced trophallaxis frequency in pesticide exposed colonies.

Similarly, antennation behavior, which serves as a key element of colony coordination and task allocation, was severely impacted by pesticidal exposure. Palmer-Young et al. [68] reported that cholinergic pesticides may impair sensory neuron function, reducing responsiveness to social cues. In the present study, reduced antennation frequency in B1–B3 colonies suggests altered social responsiveness rather than complete loss of social function. These results are in line with our findings, which revealed significantly reduced antennation in B1-B3 colonies. These treatments also had significant effects on both fecundity and adult bee lifespan. The control group had the greatest fecundity and longevity, while B1 and B2 had a sharp decrease in these tested parameters. These findings were consistent with Wu et al. [24], who reported that bees reared on pesticide-contaminated combs had reduced lifespans. Wu-Smart and Spivak [69] also documented reduced queen fecundity and reduced worker lifespan with a long-term neonicotinoid exposure. Although no statistically significant differences were detected in hatching period, larval duration, or pupal period, slight numerical reductions in treated colonies (notably A2) may indicate subtle physiological stress rather than overt developmental failure, as proposed by Rondeau et al. [70]. Thus, these findings reinforce that the continuous exposure of sub-lethal levels of insecticides gradually affects the functional capacity of the bee colonies.

## 5. Conclusions

Conclusively, it is stated that current experiments conducted under apiary field conditions might be influenced by unavoidable environmental stressors that cannot be fully controlled. However, field conditions were intentionally selected to reflect realistic exposure scenarios to enhance the ecological relevance of the findings. The current experiments demonstrate that the trophic exposure via contaminated diet to sub-lethal doses of bifenthrin and acetamiprid negatively influences the key colony performance traits of *A. mellifera*, including foraging activity, social behavior, and longevity, in a dose-dependent manner. Higher concentrations (B1, B2, A1, A2) caused more pronounced behavioral and survival impairments, whereas the lower doses (A3, A4) produced moderate effects. Reduced foraging, fewer outgoing/returning bees, and reduced pollen collection were indications of impaired orientation, likely due to neurotoxic effects. Reductions in trophallaxis and antennation further suggested impaired social feeding and social integration. Drastic declines in fecundity and adult longevity also reflected physiological stress, and small changes in brood development may indicate more subtle developmental effects. Overall, the outcomes of this study emphasized that the prolonged ingestion of pesticide-contaminated food, even at sub-lethal levels, can gradually disrupt fundamental colony functions and social stability. This underlines the critical need to consider trophic pesticidal exposure pathways in pollinator risk evaluations. This is particularly concerning for agro-ecosystems reliant on pollination, as continued pesticide exposure may jeopardize the long-term sustainability of *A. mellifera* populations and their ecological services. Therefore, future studies should focus on integrating molecular and physiological biomarkers to link behavioral changes with underlying mechanisms, validating pollen sources collected by foragers through palynological analysis, expanding experiments to multiple crops and regions, evaluating pesticide mixtures and field-relevant exposure scenarios, and employing automated or longer-term behavioral monitoring to reduce subjectivity. Long-term field trials conducted over several seasons are also recommended to better understand chronic and cumulative effects.

## Figures and Tables

**Table 1:** Description of treatments, dose preparations, and insecticide amounts (mL) applied per 500 m^2^ area for trophic toxicity assessment of Bifenthrin 10 EC and Acetamiprid 20 SP against *A. mellifera* (*n* = 3 colonies per treatment).

Treatments	Dose Preparation	Insecticide Amount (mL/500 m^2^)
B1	1/2 of the recommended dose of Bifenthrin	25 mL
B2	1/4 of the recommended dose of Bifenthrin	12.5 mL
B3	1/10 of the recommended dose of Bifenthrin	5 mL
B4	1/20 of the recommended dose of Bifenthrin	2.5 mL
A1	1/2 of the recommended dose of Acetamiprid	100 mL
A2	1/4 of the recommended dose of Acetamiprid	50 mL
A3	1/10 of the recommended dose of Acetamiprid	20 mL
A4	1/20 of the recommended dose of Acetamiprid	10 mL
T0	No Insecticide	0 mL

**Table 2:** Effects of different insecticide treatments on fecundity, hatching period, larval period, pupal period, and adult bee lifespan of *Apis mellifera* colonies under field conditions (*n* = 3 colonies per treatment). Values were expressed as mean ± SE. Different superscript letters within the same column indicate statistically significant differences among treatments based on LSD test at α = 0.05.

Treatments	Fecundity	Hatching Period	Larval Period	Pupal Period	Adult Bee Lifespan
T0	250.00 ± 12.10 ^a^	3.00 ± 0.00 ^a^	6.00 ± 0.00 ^a^	12.00 ± 0.00 ^a^	41.67 ± 0.33 ^a^
B1	169.33 ± 8.37 ^d^	2.33 ± 0.33 ^a^	5.33 ± 0.33 ^ab^	10.67 ± 0.88 ^a^	36.33 ± 0.88 ^d^
B2	183.67 ± 9.70 ^cd^	2.67 ± 0.33 ^a^	5.67 ± 0.33 ^ab^	10.67 ± 0.33 ^a^	36.33 ± 0.33 ^d^
B3	180.00 ± 9.54 ^cd^	3.00 ± 0.00 ^a^	5.67 ± 0.33 ^ab^	11.00 ± 1.00 ^a^	39.00 ± 0.58 ^bc^
B4	188.33 ± 10.17 ^bcd^	3.00 ± 0.00 ^a^	5.67 ± 0.33 ^ab^	11.33 ± 0.67 ^a^	41.33 ± 0.67 ^a^
A1	188.33 ± 7.51 ^bcd^	2.67 ± 0.33 ^a^	5.67 ± 0.33 ^ab^	10.67 ± 0.33 ^a^	37.67 ± 0.33 ^cd^
A2	194.67 ± 9.13 ^bcd^	2.67 ± 0.33 ^a^	5.00 ± 0.00 ^b^	10.67 ± 0.67 ^a^	38.67 ± 0.88 ^c^
A3	203.67 ± 11.78 ^bc^	2.33 ± 0.33 ^a^	5.67 ± 0.33 ^ab^	11.00 ± 0.58 ^a^	39.33 ± 0.33 ^bc^
A4	214.00 ± 11.24 ^b^	3.00 ± 0.00 ^a^	6.00 ± 0.00 ^a^	12.00 ± 0.00 ^a^	40.67 ± 0.67 ^ab^
F value	5.6	1.25	1.29	0.85	11
*p* value	<0.01	0.3276	0.308	0.5705	<0.01

## Data Availability

The original contributions presented in this study are included in the article. Further inquiries can be directed to the corresponding authors.
